# Wildlife Ungulate Rescue and Emergency Services in the Pisa Area (Tuscany, Italy): Evaluation of a 9-Years Period (2010–2018)

**DOI:** 10.3389/fvets.2020.00626

**Published:** 2020-09-09

**Authors:** Maria Irene Pacini, Francesca Bonelli, Angela Briganti, Simonetta Citi, Stefania Perrucci, Roberto Amerigo Papini, Micaela Sgorbini

**Affiliations:** ^1^Department of Veterinary Sciences, University of Pisa, Pisa, Italy; ^2^Department of Veterinary Sciences, Veterinary Teaching Hospital, San Piero a Grado, University of Pisa, Pisa, Italy

**Keywords:** wildlife, ungulates, trauma, car accident, roe deer, fallow deer, wild boar

## Abstract

**Background:** We analyzed the clinical data of wildlife ungulates admitted for emergency care to the Veterinary Teaching Hospital (VTH), Department of Veterinary Medicine, University of Pisa over a 9-years period.

**Methods:** Clinical data of all the wildlife ungulates admitted to the VTH were recorded. Blood samples were also taken from the animals for hematological and biochemical analysis. An assessment of ecto- and endoparasites was carried out, diagnostic imaging assessment was performed, and the outcomes were recorded.

**Results:** Data concerning clinical parameters, blood work, parasitological analysis, and diagnostic imaging diagnosis were expressed as prevalence.

**Conclusion:** The rescue and emergency treatments were related mostly to traumas caused by car accidents, followed by other causes. The traumatic injuries were mostly severe, characterized by multiple lesions involving hard and soft tissues. In this study, traffic accidents were the main cause of wildlife rescue and emergency management. This is probably due to the increased population of ungulates over the years, along with the considerable anthropization of the Pisa area.

## Introduction

In the last few decades, the wildlife population of ungulates in Italy has increased (9.1–36.2%), and the central and northern areas of Italy are the most populated. The increase is mainly due to the reduction in agricultural practices in hilly and mountainous areas, an increase in woodland and forest areas, and a reduction in livestock husbandry especially sheep and goat farming, together with a warm climate (1).

Of the total population in Italy, Tuscany has the highest prevalence of roe deer (33%) and fallow deer (51%) and the highest number of hunted wild boar (2).

Although Tuscany is characterized by wide rural areas, wild habitats are interrupted by an extensive network of roads and urban areas. Conflicts between wild ungulates and human activities are thus increasing, including serious losses to crops, with severe costs for human health along with socio-economic complications (1, 3–5). These conflicts are related to animal-vehicle collisions or entrapments in nets or similar or combine harvester injuries.

The number of wild animals rescued has been consistently increasing over the last few years (6) along with the management of injured animals. In addition, Italian law on the protection and welfare of wildlife states that people who find wild animals in difficulty must inform the local municipality. This is considered as the first mandatory step in the rescue procedure. Regional laws obligate the hospitalization of the injured or diseased animals at specialized recovery centers or veterinary services that provide medical care. Lastly, the laws on road safety make it mandatory for road users, in the event of an accident involving farm animals, pets, or protected animals, to stop and take the appropriate measures to ensure the timely rescue intervention to remove the animals from the roads.

The Veterinary Teaching Hospital (VTH) of the Department of Veterinary Sciences of the University of Pisa has taken care of the primary health care of wild animals rescued in the province of Pisa since 2010. This service is financially supported by the regional government of Tuscany (Department of Agriculture and Rural Development, Hunting and Fishing, Tuscany Region, Italy) in accordance with Italian law. The VTH provides a 24-h emergency service for wildlife ungulates, large carnivores, badgers, and porcupines that are found injured. This paper reports the clinical data, along with laboratory and parasitological findings, diagnostic imaging evaluations, and outcomes of a cohort population of roe deer, fallow deer, and wild boar admitted to the VTH over a 9-years period (2010–2018).

## Materials and Methods

Retrospective data were collected from June 2010 to December 2018. Animals were divided into four groups according to their age: (1) Group A (“newborn”): <3 months of age; (2) Group B (“small”): 3–11 months of age; (3) Group C (“young”): 1–2 years of age; (4) Group D (“adult”): >2 years of age. We have used the age categorization previously proposed by the Institute for Environmental Protection and Research (Istituto Superiore per la Protezione e la Ricerca Ambientale, ISPRA) adding a further group (Group A) in order to better assess clinical and anamnestic data. These animals are still suckling and have different behavioral characteristics and specific health problems in comparison to older ones (7–10).

Usually the animals were brought to the VTH by a voluntary association dealing with the rescue of wild and domestic injured animals. The volunteers, contacted by the people who found the animal or by the local authorities, transported the ungulates inside cages in vehicles approved for animal transport. However, in some cases the animals were brought directly by the people who found them, in which case the animals were usually tied with ropes and transported in private cars.

Data on the area and time of rescue and the history (if known) were collected by the veterinarian at admission to the VTH from the people who had rescued and transported the ungulates (voluntary associations, citizens, local authorities).

The study was carried out on injured animals admitted to the Veterinary Teaching Hospital; thus, blood collection and diagnostic procedures were carried out as part of the routine diagnostic procedures performed on the animals.

The ungulates were submitted to a clinical examination that lasted only a few minutes. If the animals were difficult to handle, the clinical examination was performed under sedation or general anesthesia to reduce the stress and handling time. Deep sedation was obtained in roe deer and fallow deer by the association of dexmedetomidine 8 ± 1.3 mcg/kg, ketamine 2 mg/kg and midazolam 0.2 mg/kg given intramuscularly. During sedation, mask oxygen was administered to all the subjects. Induction of general anesthesia was performed using propofol 2 mg/kg IV, and endotracheal intubation was performed only in the case of respiratory depression. In wild boar, deep sedation was performed with dexmedetomidine 15 mcg/kg, ketamine 0.2 mg/kg, and methadone 0.2 mg/kg given intramuscularly using the same syringe. Animals not submitted to euthanasia after the clinical and radiological assessments were injected with atipamezole 70–100 mcg/kg IM, depending on the dose of dexmedetomidine received.

During the clinical examination, the state of nutrition of the animals was assessed. Body condition score (BCS) was evaluated in roe deer and fallow deer using a score ranging from 1 (lean) to 5 (fat), as previously reported (11). We evaluated the BCS of the wild boar referring to scores used for pigs ranging from 1 to 5 (12).

When possible, during the clinical examination, fecal and blood samples were collected. Blood samples were taken from the jugular or cephalic vein and collected in EDTA (Ethylenediaminetetraacetic acid) and heparinized tubes. The EDTA samples were processed within 15 min of collection for a complete blood count (CBC), using an automated cell counter (Lasercyte, Idexx, USA), and assessing the following parameters: red blood cell count (RBC), white blood cell count (WBC), hemoglobin (Hgb), haematocrit (Hct), mean corpuscular volume (MCV), mean corpuscular hemoglobin (MCH), mean corpuscular hemoglobin concentration (MCHC), and platelet count (PLT). The results obtained have been compared with the reference ranges reported in literature (13–15).

Heparinized samples were centrifuged at 2,100 rpm for 10 min and plasma was used to assess the following parameters using an autoanalyzer (Liasys, Analyzer Medical System-AMS, Rome, Italy); creatinine (kinetic modified Jaffè method) and urea (kinetic enzymatic method), total bilirubin (colorimetric method without DMSO concentrations), aspartate aminotransferase (AST) (kinetic method UV—IFCC), alanine transaminase (ALT), gamma glutamyl-transferase (GGT) (kinetic method-Szasz-Tris), creatine kinase (CK) (kinetic method UV) and alkaline phosphatase (ALP) (kinetic method) activities. The results obtained have been compared with the reference ranges reported in literature (13, 15–18).

Fecal samples were collected, stored at +5°C, and parasitological analyses were carried out within 12 h of sample collection. Specimens were examined microscopically using the passive flotation technique (19) with a saturated solution of sodium chloride (specific gravity = 1.20) as flotation fluid to detect coccidian oocysts and nematode eggs. The parasite burden was estimated by determining the number of oocysts per gram (OPG) and eggs per gram (EPG) of tools. Counts were performed by the modified McMaster method (20) with the lowest detection limit of 50 OPG/EPG per gram. In addition, deer fecal samples were examined by the Baermann technique to detect bronchopulmonary nematode larvae (21). Samples were weighted, and the number of larvae per gram (LPG) of feces was calculated. Mean and range of OPG, EPG and LPG were determined.

At the initial examination, ectoparasites (ticks, lice, hippoboscids) were detected by close visual inspection of the hair and skin over different regions of the animals for a maximum time of 15 min. Close attention was paid to the following sites: ears, neck and flanks. For each type of ectoparasite found, between one and ~20 specimens were collected from the same animal even if more specimens were present on the host. Ectoparasites were promptly removed using fingers or tweezers, collected in sterile tubes, separated by host species, kept in 70% ethyl alcohol, and microscopically examined within 24 h. The different numbers of females, males, immature stages, and adults were not calculated for this study. When mange was suspected, diagnosis was made by deep skin scrapings. The ectoparasite burden score was not calculated. All the parasitic agents detected were microscopically identified by their morphological characteristics using standard taxonomic keys reported in reference textbooks (22, 23).

A radiological examination was performed when the animal showed orthopedic and/or neurological signs or when, according to the anamnestic data, the animal had possibly been involved in severe trauma. The x-ray exams were analyzed by an experienced radiologist.

The laboratory work, parasitological exams and diagnostic imaging performed were decided on by the veterinarian who admitted the animal to the VTH based on the assessment of clinical data.

Proportions were calculated for all the data collected.

## Results

A total of 186 animals were rescued: 148/186 (79.6%) roe deer (*Capreolus Capreolus*), 19/186 (10.5%) fallow deer (*Dama dama)*, and 19/186 (10.5%) wild boar (*Sus scrofa)*. Results concerning the species and sex divided by age (Group A, Group B, Group C, and Group D) are reported in Table 1.

**Table 1:** Number of rescued animals presented by age category, species, and sex.

**Age category^**a**^**	**Roe deer**		**Fallow deer**		**Wild boar**		**Total**
	**Male**	**Female**	**Male**	**Female**	**Male**	**Female**	
Group A	13	17	-	-	4	6	40
Group B	6	7	1	1	3	3	21
Group C	21	24	4	3	1	1	54
Group D	34	26	6	4	-	1	71
Total	148		19		19		186

a *Group A: <3 months of age; Group B: 3–11 months of age; Group C: 1–2 years of age; Group D: >2 years of age*.

Based on the reason for hospital admission, 131/186 (71%) animals had been injured in car accidents and 16/186 (8.6%) by a combine harvester, 5/186 (2.7%) had been accidentally trapped in nets or fences, 4/186 (2.1%) showed signs of severe respiratory disease, 5/186 (2.7%) had been bitten by domestic dogs, and 25/186 (13.4%) were victims of an ill-advised rescue because the people rescuing the animals believed that the fawns/piglets had been abandoned by their mothers. The reason for hospital admission based on the age categories are reported in Table 2.

**Table 2:** Reasons for admission of rescued animals to the Veterinary Teaching Hospital.

**Reasons for hospital admission**	**Group A^**a**^**	**Group B^**a**^**	**Group C^**a**^**	**Group D^**a**^**	**Total *n*/%^**b**^**
Injured in car accidents	1	12	49	69	131/70.4%
Injured by a combine harvester	16	-	-	-	16/8.6%
Accidentally trapped in nets or fences	-	3	2	-	5/2.7%
Bitten by domestic dogs	-	4	1	-	5/2.7%
Respiratory diseases	-	-	2	2	4/2.2%
Victims of an ill-advised rescue	23	2	-	-	25/13.4%
Total	40	21	54	71	186

*Results are presented according to age categories*.

a *Group A: <3 months of age; Group B: 3–11 months of age; Group C: 1–2 years of age; Group D: >2 years of age*.

b *Number and proportions of rescued animals according by reasons for admission*.

During hospitalization, 143/186 (76.8%) animals died or were humanely euthanized, whereas 43/186 (23.2%) were released.

Roe deer from group A were rescued only during spring, while animals from groups B, C, and D were rescued mostly during both spring and summer. All the roe deer were found in hilly areas located south/southwest of the Arno river. As with the roe deer, the fallow deer were rescued mostly in the spring and summer and were always found in a small area peripheral to the “San Rossore, Migliarino, Massaciuccoli” natural park. Wild boars were rescued throughout the entire study period and throughout the district.

### Body Condition Score (BCS)

All animals included in the study showed a BCS ranging between 2.5/5 and 3/5. Only two roe deer and one fallow deer with clinical signs of respiratory diseases had lower BCS values (1.5/5, 2/5, and 2/5, respectively).

### Hematology and Clinical Chemistry

#### Roe Deer

A CBC was performed in 31/148 (21%) roe deer and biochemical analyses in 35/148 (23.7%).

Six out of 31 (19.4%) roe deer, 5/6 subjects from group B and 1/6 from group D, showed a lower PCV value (18.1–42.2%) than the reference interval (43–59%) and a lower RBC value (4.1–8.6 M/μl) (reference interval: 9.6–13.8 M/μl). All of these had been affected by traumas.

Leukocytosis was observed in 2/31 (6.5%) animals (reference interval: 2.8–8.1 K/μl). In particular, 1/2 roe deer from group C (10.8 K/μl) showed clinical signs of respiratory disease, and 1/2 of group D (10.0 K/μl) had been injured in a car accident.

Compared to the reference range (976 ± 599 U/L), increased CK plasmatic activity was detected in 19/35 (54.3%) animals (1,617–166,960 U/L), whereas 18/35 (51.4%) showed increased AST activity (327–3,653 U/L) (reference range: 180 ± 108 U/L). Both plasmatic urea (reference range: 18 ± 10 mg/dl) and creatinine concentrations (reference range: 1.49 ± 0.04 mg/dl) were increased in 18/35 (51.4%) (44–254 mg/dl) and 14/35 (40%) (2.20–4.50 mg/dl) roe deer from group D, respectively.

#### Fallow Deer

The bloodwork was carried out in 7/19 (36.8%) fallow deer of group D, of which 6/19 (31.6%) had been injured in car accidents and 1/19 (5.3%) showed signs of respiratory diseases. No CBC abnormalities were detected in any of the seven animals, while increased CK (1,013–77,090 U/L) (reference range: 368.8 ± 330 U/L) and AST (378–4,230 U/L) (reference range: 95.2 ± 82.0 U/L) activities were detected in all of the subjects assessed. Increased plasmatic ALP activity (reference range: 113.9 ± 64.0 U/L) was observed in 2/19 (10.5%) (267 and 1,343 U/L) fallow deer, which had both been injured in a car accident.

#### Wild Boar

Hematological and biochemical analyses were carried out in 2/19 (10.5%) animals, both belonging to group B. In these subjects, only CK (14,680–142,320 U/L) (reference range: 303–9,869 U/L) and AST (333–1,466 U/L) (reference range: 19–161 U/L) activities were high in both animals evaluated.

### Parasitological Examination

#### Roe Deer

Parasitological examinations were carried out in 83/148 (56.1%) roe deer, 16/83 of group B and 67/83 of groups C+D. Overall, 80/83 (96.4%) roe deer were found to be coprologically positive for endoparasites by the flotation technique. In particular, 37/83 (44.6%), 2/37 of group B and 35/37 of groups C+D, were positive for gastrointestinal strongyle eggs, 32/83 (38.5%), 13/32 of group B and 19/32 of groups C+D, for *Eimeria* spp. oocysts and 11/83 (13.2%), all in groups C/D, for both. The results of the Baermann method revealed numerous nematode larvae consistent with first stage larvae of *Varestrongylus capreoli* in 10/83 (12%) animals, all of groups C+D. The parasitological results are shown in Table 3.

**Table 3:** Number and proportions (*n*/%) of roe deer positive for endoparasites by coprological analyses.

**Endoparasites**	**Group B^**a**^**	**Group C+D**^**a**^		**Total**
	***n*^**b**^ = 16**	***n***^**b**^ **=** **67**		***n*^**b**^ = 83**
	***n*/% positive**	***n*****/% positive**		***n*/% positive**
Gastrointestinal strongyle eggs (EPG^*c*^)	2/12.5%	35/52.2%	11/16.4%	37/44.6% Mean: 5,050 Range: 1,700–6,650
*Eimeria* spp. (OPG^*d*^)	13/81.3%	19/28.4%		32/38.6% Mean: 3,150 Range: 650–7,800
*Varestrongylus capreoli* (LPG^*e*^)	-	10/14.9%		10/12.1% Mean: 600 Range: 50–2,200

*Results are presented according to age categories*.

a *Group A: <3 months of age; Group B: 3–11 months of age; Group C: 1–2 years of age; Group D: >2 years of age*.

b *Number of animals tested*.

c *Eggs per gram*.

d *Oocysts per gram*.

e *Larvae per gram*.

Ectoparasites were collected in animals of groups C+D: the hard tick Ixodes ricinus were found in 46/67 (68.7%) animals, the chewing louse *Damalinia* (Cervicola) meyeri in 12/67 (17.9%), and the deer ked *Lipoptena cerv*i in 20/67 (29.9%).

#### Fallow Deer

Parasitological examinations were carried out in 13/19 (68.4%) fallow deer, 3/13 of group C and 10/13 of group D. Overall, 8/13 (61.5%) fallow deer, all of group D, were found to be coprologically positive for both *Eimeria* spp. oocysts (mean = 1,650 OPG, range = 100–4,250 OPG) and gastrointestinal strongyle eggs (mean = 2,500 EPG, range = 1,500–3,500 EPG). Ectoparasite infestation with both *I. Ricinus* and *L. cervi* occurred in 9/13 (69.2%) fallow deer of group D.

#### Wild Boar

Parasitological examinations were performed in 8/19 (42.1%) wild boar, 3/8 of group A and 5/8 of group B. The results of the floatation technique revealed mixed infections with coccidia oocysts (mean = 3,100 OPG, range = 150–6,700), *Ascaris suum* eggs (mean = 750 EPG, range = 150–3,000 EPG) and *Metastrongylus* spp. eggs (mean = 400 EPG, range = 50–1,400 EPG) in all the animals examined. Diffuse alopecia and lichenification associated with moderate pruritus were observed in 3/8 (37.5%) wild boars of group A. Microscopic examination of skin scrapings revealed the presence of live and actively motile mites consistent with *Sarcoptes scabiei* infection.

### Radiology

#### Roe Deer

A total of 101/148 roe deer (68.2%) underwent radiological examination. The diagnostic imaging revealed lesions in 93/101 (92.1%) roe deer, while no abnormalities were present in 8/101 (7.9%) of the animals.

Lesions were diagnosed as follows: front limb fractures/dislocations in 12/101 (11.9%) animals; hind limb fractures/dislocations in 43/101 (42.6%). One/101 roe deer of group C (0.9%) was affected by septic arthritis (cytological examination and bacteriology).

Skull fractures were diagnosed in 3/101 (2.9%) animals; 2/101 (1.9%) roe deer of group D showed rib fractures; 10/101 (9.9%) showed vertebral fractures and/or dislocation. Multiple pelvis fractures were diagnosed in 27/101 (26.7%) roe deer. A total of 16/101(15.8%) animals showed more than one fracture. In 1/101 (0.9%) of group B, a neurological bladder was present in association with a vertebral luxation.

Internal lesions were observed in 18/101 (17.8%) as follows: pulmonary contusions in 8/101 (7.9%) animals (2/8 associated with rib fractures), abdominal and/or thoracic effusion in 16/101 (15.8%) roe deer.

The results divided by age categories are reported in Table 4.

**Table 4:** Number (*n*) of positive radiological findings in roe deer.

**Radiological findings**	**Group A^**a**^*n*^**b**^ = 12**	**Group B^**a**^*n*^**b**^ = 11**	**Group C^**a**^*n*^**b**^ = 29**	**Group D^**a**^*n*^**b**^ = 49**	**Total *n*^**b**^ = 101 *n*/%^**c**^**
	***n*** **of positive radiological findings**				
Front limb fractures/dislocations	2	1	5	4	12/11.9%
Hind limb fractures/dislocations	6	5	11	21	43/42.6%
Septic arthritis	-	-	1	-	1/0.9%
Skull fractures	-	1	2	-	3/2.9%
Rib fractures	-	-	-	2	2/1.9%
Vertebral fractures/dislocations	-	3	2	5	10/9.9%
Multiple pelvis fractures	-	7	11	9	27/26.7%
Pulmonary contusion	1	2	4	1	8/7.9%
Abdominal/thoracic effusion	-	4	6	6	16/15.8%

*Results are presented according to age categories*.

a *Group A: <3 months of age; Group B: 3–11 months of age; Group C: 1–2 years of age; Group D: >2 years of age*.

b *Number of animal tested*.

c *Number and proportions of animals positive for radiological lesions*.

#### Fallow Deer

At admission, 16/19 (84.2%) fallow deer underwent a radiological examination. The radiological lesions were diagnosed only in animals injured by vehicles (13/16, 81.2%), while no abnormalities were present in the other three fallow deer.

The radiological lesions diagnosed were fractures to the maxillary bone in 1/16 (6.3%) of group C animals, front limb or hind limb fractures in 7/16 (43.7%), rib fractures in 1/16 (6.3%) of group C, pelvis fractures in 2/16 (12.5%) of group D, and vertebral bone fractures in 2/16 (12.5%) fallow deer. In 4/16 (25%) animals, severe pulmonary contusion was also diagnosed in association with bone fractures. The results divided by age categories are reported in Table 5.

**Table 5:** Number (*n*) of positive radiological findings in fallow deer.

**Radiological findings**	**Group B^**a**^*n*^**b**^ = 2**	**Group C^**a**^*n*^**b**^ = 5**	**Group D^**a**^*n*^**b**^ = 9**	**Total *n*^**b**^ = 16 *n*/%^**c**^**
	***n*** **of positive radiological findings**			
Limb fractures/dislocations	1	1	5	7/43.8%
Maxillary bone fractures	-	1	-	1/6.3%
Rib fractures	-	1	-	1/6.3%
Vertebral fractures	1	1	-	2/12.5%
Multiple pelvis fractures	-	-	2	2/12.5%
Pulmonary contusion	-	3	1	4/25%

*Results are presented according to age categories*.

a *Group A: <3 months of age; Group B: 3–11 months of age; Group C: 1–2 years of age; Group D: >2 years of age*.

b *Number of animals tested*.

c *Number and proportions of animals positive for radiological lesions*.

#### Wild Boar

Three out of 19 (15.8%) wild boar underwent radiological examination which revealed multiple fractures caused by a car accident. In particular, 1/3 of group B had a fracture of the humerus, 1/3 of group B had a fracture of the humerus and a tarsal-metatarsal dislocation, and 1/3 of group C had multiple fractures of the pelvis and lumbar vertebrae. The results divided by age categories are shown in Table 6.

**Table 6:** Number (*n*) of positive radiological findings in wild boars.

**Radiological findings**	**Group B^**a**^*n*^**b**^ = 2**	**Group C^**a**^*n*^**b**^ = 1**	**Total *n*^**b**^ = 3 *n*/%^**c**^**
	***n*** **of positive radiological findings**		
Homerus fracture	2	-	2/66.7%
Tarsal/metatarsal dislocation	1	-	1/33.3%
Vertebral fractures	-	1	1/33.3%
Multiple pelvis fractures	-	1	1/33.3%

*Results are presented according to age categories*.

a *Group A: <3 months of age; Group B: 3–11 months of age; Group C: 1–2 years of age; Group D: >2 years of age*.

b *Number of animals tested*.

c *Number and proportions of animals positive for radiological lesions*.

### Outcome

The animals released or whose custody was given to authorized individuals or wildlife recovery centers consisted of neonates that had been inappropriately taken by people or those with mild to moderate soft tissue lesions. On the other hand, the animals that underwent euthanasia had been affected by severe or generalized lesions which had poor prognosis for recovery: for example, fracture of the spinal cord with paresis or paralysis, fracture of the forelimbs, hindlimbs or pelvis that needed long-postoperative management and immobilization, or neurological signs not responsive to treatment.

A total of 43/186 (23.11%) animals were released into their habitat after a complete recovery, while 143/186 (76.9%) died or were euthanized. The results divided by species and age category are presented in Table 7.

**Table 7:** Animal rescue outcomes.

**Outcome**	**Roe deer**				**Fallow deer**			**Wild boar**				**Total**
	***n*****/%**^**b**^				***n*****/%**^**b**^			***n*****/%**^**b**^				***n*/%^**b**^**
Released	Group A^a^	Group B^a^	Group C^a^	Group D^a^	Group B^a^	Group C^a^	Group D^a^	Group A^a^	Group B^a^	Group C^a^	Group D^a^	43/23.1%
	14	4	7	6	1	2	-	7	2	-	-	
	31/20.9%				3/19.8%			9/47.4%				
Died/ humanely euthanized	Group A^a^	Group B^a^	Group C^a^	Group D^a^	Group B^a^	Group C^a^	Group D^a^	Group A^a^	Group B^a^	Group C^a^	Group D^a^	143/76.9%
	16	9	28	64	1	5	10	3	4	2	1	
	117/ 79.1%				16/ 84.2%			10/ 52.6%				

*Results are presented according to species and age categories*.

a *Group A: <3 months of age; Group B: 3–11 months of age; Group C: 1–2 years of age; Group D: >2 years of age*.

b *Number and proportions of animals divided according by outcome*.

#### Roe Deer

A total of 31/148 (20.9%) roe deer were released into their habitat after a complete recovery. Of these, 14/31 were from group A, 4/31 from group B, 7/31 from group C, and 6/31 from group D. A total of 117/148 (79.1%) roe deer died or were euthanized. Of these, 16/117 were under 3 months, 9/117 were from group B, 28/117 were from group C, and 64/117 from group D.

#### Fallow Deer

Three out of 19 (15.8%) fallow deer, 1/3 of group B and 2/3 of group C, survived and were released into the wild, while 16/19 (84.2%) died or were euthanized.

#### Wild Boar

Nine out of 19 (47.4%) animals, 7/9 of group A and 2/9 of group B, were given to authorized individuals, while 10/19 (52.6%) died or were euthanized.

## Discussion

This paper reports the data collected during a 9-years period on wildlife ungulates by rescue and emergency services in the province of Pisa.

In our study, the rescues and hospitalization of the ungulates were mostly due to road accidents (71%) with a constant prevalence over the years. This is in line with a previous report (3) according to which collisions between wild animals and vehicles frequently occur in Tuscany and the province of Pisa. Wildlife road accidents were frequently found to occur on municipal roads (24, 25) which is in agreement with our findings. Most of the roe deer, fallow deer and wild boar included in our study were found along secondary roads. Municipal roads that cross rural areas (3, 4, 24) are characterized by little traffic and few road barriers; thus, wild animals can cross easily (26–28). In general, wildlife-vehicle collisions are a growing problem worldwide, with millions of accidents with large mammals occurring every year (29). Car collisions usually cause severe injuries and prevent the animal from running away from the impact site, thus the rescues are more frequent.

Regarding roe deer, the number of rescues following a car accident was higher between April and August. This is because roe deer move much more during these months than the rest of the year. In the spring, yearlings move away from the family group searching for new territories, and the summer is the mating season (1, 3). Fallow deer injured by cars, on the other hand, were mostly hospitalized in the summer, when these animals move to richer pastures (24). In contrast, wild boar involved in car accidents were rescued throughout the whole year.

In our study, the number of rescues of roe deer (73%) and fallow deer (90%) due to car collisions was higher than the findings for wild boar (33%), in contrast with previous reports (2, 9, 24). Data collected between 2001 and 2009 in Tuscany showed a higher number of car accidents caused by wild boar compared to roe deer (1,335 vs. 1,222) (3, 24). This difference could be related to the more severe and expensive damage that wild boar cause compared to other wild animals. The official report (24) revealed that the number of requests for damage compensation related to road accidents with wild animals was greater for wild boar than other species. Our data reported the number of rescues not related to requests for compensation and showed that roe deer were hospitalized more frequently than wild boar. The hypothesis supporting our findings could be that roe deer are more light-weight and less robust than wild boar, thus they may be more vulnerable to the impact of cars and incur more serious lesions. In addition, handling and transporting deer is generally easier than for wild boar.

Combine harvesters are the main cause of rescue for newborn roe deer. In fact, 16/30 (53.3%) newborn roe deer had been injured by these agricultural machines, and 15/16 (93.7%) were euthanized due to the severity of the lesions. This may happen because in spring the female roe deer give birth to newborns which they hide to limit the predation in the fields during the first period of suckling (30–33). According to previous studies, the prevalence of dead fawns during harvesting ranges from 5 to 26% (30, 31, 34, 35) or, in absolute terms, from 1,500 head per year in Switzerland to 84,000 head per year in Germany (30). Our data showed a higher incidence of injuries caused by agricultural machines. This might be due to the different harvesting periods in Southern Europe compared to North European countries. In Italy, harvesting corresponds with the peak period of roe deer births, while in northern countries, the higher prevalence of births is at the end of June; thus, the loss of fawns in Northern Europe is lower than in our region (30–32).

In this report, 13.4% of the total number of wild animals rescued (13/25 roe deer fawns and 12/25 wild boar neonates) were taken by hikers who believed that the newborns were orphans. These newborns were the subjected to unwarranted rescue due to the rescuers lack of knowledge and training. Only 15/25 neonates, 75% (9/12) of wild boar and 46% (6/13) of roe deer, survived to be released. Hand-rearing a wild ungulate is usually challenging and requires knowledge of the animal's biology, and thus has a very low success rate (36–38). The hand-rearing methodology for each species is unique and providing an appropriate milk substitute is one of the most important factors (36, 38, 39).

Roe deer fawns are particularly difficult to hand-rear and the prognosis for fawns brought into captivity is poor (37, 38). It has been reported that the success rate of hand-rearing newly born fawns that have not yet consumed milk is close to zero and that of a 1-week old fawn is 50% (37). Moreover, most of the neonates were taken to the VTH several hours or even days after the rescue, when the clinical conditions had become very critical. Often people took the neonates home first and tried to feed them using inappropriate milk and incorrect procedures. The administration of inappropriate milk substitutes can result in severe diarrhea, dehydration, stunted growth, and also death (37, 40). For example, roe deer milk contains more fat, protein, and minerals than cow's milk, which is deficient in essential nutrients and can lead to diarrhea (37).

A total of 2.7% of the wild ungulates rescued (four roe deer and one fallow deer) were found trapped in nets or fences and 2.7% had been wounded by privately-owned dogs. This might be because ungulates approach residential areas. In recent decades, the wildlife population of ungulates in Italy has increased and populated the countryside and city suburbs abandoned by the man (1, 25, 41). Wild animals therefore sometimes go into areas where there are human activities and thus get trapped in fences or wounded by dogs (24).

Our data on radiological assessments showed a different pattern of lesions in the roe deer compared to the other species. In wild boar and fallow deer, fractures were diagnosed both on the front and hind limbs, while roe deer were affected mostly by multiple fractures on the backside (hind limbs, pelvis, lumbar vertebrae). This could be because the roe deer might have changed direction in order to run away from a car, and thus were hit in the backside, while the fallow deer and wild boars were hit while they were crossing the road. Moreover, the 16% roe deer had fractures in different parts of the body and the 27% showed multiple fractures of the pelvis, while all follow deer undergoing radiological examination had only one fracture. This could be due to the different sizes of the two species. The fallow deer is taller, heavier and more robust than the roe deer. Therefore, in the event of a car-collision, the roe deer may suffer multiple injuries, while fallow deer may be hit only on one area of the body.

Regarding the hematology and clinical chemistry, we found abnormal values both for the hemogram and biochemical parameters. In particular, the RBC and Hct of some of the injured roe deer were lower than the reference interval, probably due to blood loss following the trauma, more than anemia related to an undiagnosed chronic disease or a food deficiency (42–45).

Neutrophilic leukocytosis was found in two subjects. One animal showed signs of pneumonia at the clinical examination, thus the altered hemogram values could be due to the ongoing lung infection. The other animal had been traumatized and the altered values could be a sign of a stress leukogram caused by glucocorticoid release following the stressful event.

The increase in CK and AST activities are likely to be associated with rhabdomyolysis due to trauma or muscle exhaustion due to incorrect transport practices. In fact, some of the ungulates were transported to the VTH tied with ropes. The restraint during clinical examination was minimal and lasted a few minutes and difficult-to-contain animals were sedated.

Increased concentrations of urea and creatinine were also found. This could be due to dehydration and secondary pre-renal insufficiency and/or renal insufficiency caused by tubular necrosis due to the release of myoglobin during rhabdomyolysis (46–49).

Coprological examinations showed several gastrointestinal and respiratory parasites in all the species rescued. However, the prevalence and burden estimation of the identified parasites are difficult to compare with previous investigations in Italy, where sampling was performed on necropsied animals directly from the viscera (50, 51). The state of nutrition was excellent in most of the subjects admitted to the hospital, despite the presence of intestinal and/or bronchopulmonary parasites. The adaptation of wildlife species to parasitosis is already known (52) and supports our findings. Parasites live in a delicate balance with their host and generally tend to have mild effects on the health of the parasites with mostly long-term effects rather than causing the death of their hosts. They usually cause a gradual decrease in body conditions and fertility (52, 53). Although there are examples of parasitic infections that cause significant symptoms and lead to death, including *V. capreoli* infections, parasitism usually involves small reductions in the likelihood that a host will reproduce or survive, without obvious clinical symptoms (53, 54).

In most cases, the hospitalized animals died spontaneously or were euthanized. This was due to the severity of the traumas diagnosed and to the severe clinical abnormalities on admission to the hospital. Only 43 out of 186 animals survived and were released, of which 15/43 (13 from group A and two from group B), were healthy animals wrongly brought to the hospital and 28/43 (six from group A, nine from group B, seven from group C, and six from group D) were affected by skin and soft-tissue injuries. All animals with fractures (mostly severe) or permanent neurological damage died spontaneously or were euthanized.

## Conclusions

Traumatic injuries caused by road accidents were the main reason for the rescue and hospitalization of wild ungulates in this study. Collision with a vehicle caused life threatening injuries, mostly affecting the skeletal system. Other reasons for presentation were unwarranted rescues of neonates, injuries caused by combine harvesters, entrapments in nets or fences, bites by domestic dogs or, to a much lesser extent, diseases.

Animals suffering from lesions affecting the skeletal system and permanent neurological damage usually died or underwent euthanasia, while for ungulates suffering only soft tissue injuries, the outcome was more favorable.

Based on the knowledge gained from this study, we advocate improving people's knowledge of different wildlife species in order to reduce the number of unwarranted rescues, to improve rescue and transport procedures and to make people more careful during car journeys and harvesting.

## Data Availability Statement

The datasets analyzed in this article are not publicly available. Requests to access the datasets should be directed to Micaela Sgorbini, micaela.sgorbini@unipi.it.

## Ethics Statement

Our study was carried out on injured animals admitted to the Veterinary hospital, thus hematological and biochemical analyses, and radiological examination were performed as part of the routine diagnostic procedures. Since July 2010 the Fauna Defence Service of Tuscany has entrusted the VTH to provide a 24-h emergency service for wild animals found injured within the province of Pisa and the ethic approval of clinical procedures was implicit in this agreement.

## Author Contributions

MP, FB, and MS: conceptualization, methodology, formal analysis, investigation, writing, writing review, and editing. MS: resources, project administration, and funding acquisition. MP, FB, AB, SC, SP, RP, and MS: investigation, data processing, visualization, supervision, writing, writing review, and editing. All authors contributed to the article and approved the submitted version.

## Conflict of Interest

The authors declare that the research was conducted in the absence of any commercial or financial relationships that could be construed as a potential conflict of interest.
