# Corrigendum: Wildlife Ungulate Rescue and Emergency Services in the Pisa Area (Tuscany, Italy): Evaluation of a 9-Years Period (2010–2018)

**DOI:** 10.3389/fvets.2020.629099

**Published:** 2021-01-08

**Authors:** Maria Irene Pacini, Francesca Bonelli, Angela Briganti, Simonetta Citi, Stefania Perrucci, Roberto Amerigo Papini, Micaela Sgorbini

**Affiliations:** ^1^Department of Veterinary Sciences, University of Pisa, Pisa, Italy; ^2^Department of Veterinary Sciences, Veterinary Teaching Hospital, San Piero a Grado, University of Pisa, Pisa, Italy

**Keywords:** wildlife, ungulates, trauma, car accident, roe deer, fallow deer, wild boar

Stefania Perrucci was not included as an author in the published article. The corrected Author Contributions Statement appears below.

“MP, FB, and MS: conceptualization, methodology, formal analysis, investigation, writing, writing review, and editing. MS: resources, project administration, and funding acquisition. MP, FB, AB, SC, SP, RP, and MS: investigation, data processing, visualization, supervision, writing, writing review, and editing. All authors contributed to the article and approved the submitted version.”

Also in the original article, there were errors. In the “Materials and Methods” section, some of the parasitological methods were not correctly reported, while a few words in the “Results” section should be corrected. Moreover, a few statements in the “Discussion” need to be corrected and a reference should be added in the text of the Discussion and in the reference list.

Corrections have been made to “Materials and Methods,” page 3, left column, lines 3-7; “Results,” right column, lines 22 and 33; “Discussion,” page 7, right column, lines 19-22 and 31-35; and “References,” where a new reference, i.e., 54, should be added in the text and in the reference list:

## Materials and Methods

“In addition, deer fecal samples were examined by the Baermann technique to detect bronchopulmonary nematode larvae (21). Samples were weighted, and the number of larvae per gram (LPG) of feces was calculated.”

## Results

“spp” is replaced with “spp.”

## Discussion

“However, the prevalence and burden estimation of the identified parasites are difficult to compare with previous investigations in Italy, where sampling was performed on necropsied animals directly from the viscera (50, 51).”

“Although there are examples of parasitic infections that cause significant symptoms and lead to death, including *V. capreoli* infections, parasitism usually involves small reductions in the likelihood that a host will reproduce or survive, without obvious clinical symptoms (53, 54).”

A new reference is added (reference no. 54).

The authors apologize for these errors and state that they do not change the scientific conclusions of the article in any way. The original article has been updated.
